# Cardiovascular–Kidney–Metabolic Syndrome: Models of Care

**DOI:** 10.31083/RCM47862

**Published:** 2026-07-20

**Authors:** Isaac Chung, Dorottya I. Fricska, Suvro Banerjee, Pooja Banerjee, Victor Hugo Gomez Johnson, Nicholas M.P. Annear, Arpita Ray Chaudhury, Mimi Chen, Lisa Anderson, Debasish Banerjee

**Affiliations:** ^1^Renal and Transplantation Unit, St George’s University Hospitals NHS Foundation Trust, SW17 0QT London, UK; ^2^Department of Medicine, School of Health and Medical Sciences, City St George’s, University of London, SW17 0RE London, UK; ^3^Department of Cardiology, Apollo Multispecialty Hospitals, 700054 Kolkata, India; ^4^Department of Nephrology, Apollo Multispecialty Hospitals, 700054 Kolkata, India; ^5^Nephrology Department, Instituto Nacional de Cardiologia, Ignacio Chavez, 14080 Mexico City, Mexico; ^6^Department of Nephrology, Institute of Post Graduate Medical Education & Research (IPGMER), 700020 Kolkata, India

**Keywords:** cardiovascular–kidney–metabolic syndrome, cardiovascular diseases, clinic, multidisciplinary, interdisciplinary, kidney, risk factors

## Abstract

Cardiovascular–kidney–metabolic (CKM) syndrome represents the complex interplay between cardiovascular disease (CVD), chronic kidney disease (CKD), and metabolic disorders such as type 2 diabetes and obesity. These conditions share overlapping risk factors, pathophysiological mechanisms, and clinical outcomes, leading to progressive organ dysfunction, as well as increased morbidity and mortality. Traditional disease-specific care models, in which cardiology, nephrology, and endocrinology operate in separate clinics, are increasingly inadequate for addressing the integrated nature of CKM syndrome. Patients often experience fragmented care, with different specialties prioritizing different medications and communicating slowly via letters, which delays important guideline-directed medical therapy (GDMT) for each organ system, potentially leading to higher rates of hospital admissions and mortality. New models of care are urgently needed to improve outcomes through coordinated, patient-centered, and multidisciplinary approaches. Future integrated CKM care models should emphasize cross-specialty collaboration, data sharing, and shared decision-making to optimize treatment and deliver comprehensive care plans that consider all three specialty domains. Advances in digital health, precision medicine, and population health analytics can support these models by enabling more accurate risk stratification, personalized interventions, and continuous monitoring across the care continuum. This review, originating from an International Society of Nephrology Transplant Sister Center Meeting, synthesizes the available evidence on multispecialty clinics and other interventions designed to improve care for patients with multiple CKM comorbidities.

## 1. Introduction

Cardiovascular–kidney–metabolic (CKM) syndrome is an emerging global public health problem that disproportionately affects people in low- and middle-income countries (LMICs) [1,2]. CKM describes the interplay between metabolic diseases, such as obesity and type 2 diabetes mellitus (T2DM), chronic kidney disease (CKD), and cardiovascular disease (CVD) [3]. The risk factors for CKM syndrome encompass those of cardiorenal and cardiometabolic syndromes and, indeed, the established risk factors for CVD, CKD, and metabolic diseases. Additionally, due to the interconnectedness of the heart, kidney, and metabolic health, dysfunction in any one of these systems can adversely affect the other two, thereby increasing overall disease burden and leading to higher rates of morbidity and mortality.

CKM syndrome is classified into five stages (0–4), which facilitate diagnosis, identify areas for preventive action, and guide management [3]. Stage 0 represents optimal CKM health, without CKM risk factors or established CVD. Stage 1 includes excess adiposity, insulin resistance, or prediabetes, without other metabolic risk factors or CKD. In Stage 2, established metabolic risk factors (e.g. metabolic syndrome, high triglycerides, hypertension, diabetes) or early CKD are present. Stage 3 is characterised by early, subclinical CVD or a very high predicted risk of developing CVD or advanced CKD. Finally, Stage 4 indicates established, clinical CVD with or without ESRD within the CKM [3]. While most current treatment options address the different organ dysfunctions separately (Table 1, Ref. [4,5,6,7,8,9,10,11,12]), randomized controlled trials have identified therapies that confer benefits across all three systems, such as sodium–glucose co-transporter 2 (SGLT-2) inhibitors [13], glucagon-like peptide-1 (GLP-1) agonists [14], and non-steroidal mineralocorticoid receptor inhibitors [15]. Treatment of CKM stage 2 focuses on treating individual components of CKM, including controlling hypertension to a target of <130/80 mmHg, aggressively controlling blood glucose, achieving appropriate weight loss, and reducing albuminuria. Although syndrome-level evidence for treating CKM remains limited, there is strong evidence of risk reductions when treating the individual components that constitute CKM.

**Table 1.** **Models of care in the literature**.

Location	Model of care	Number of patients and study period	Intervention	Outcomes
Vancouver, Canada [4]	Combined clinic (C, R, M)	A total of 139 participants were randomized over 4 months, and 69 were evaluated in the combined clinic (intervention).	Face-to-face	Reduced hospital admissions
			Diabetes, cardiac, or renal nurse; dietitian, pharmacist; nephrologist; cardiologist; endocrinologist.	*after 12 months of follow-up*, with cost savings (from reduced clinic appointments) for CAD 86,400 per year.
Liverpool, UK [5]	Multispecialty MDT	Discussed 334 patients over 18 months.	Virtual meeting HF cardiologists from community, secondary, and tertiary care; HF specialist nurses from community and hospital settings; nephrologist, endocrinologist, palliative care specialist, chest physician, geriatrician, pharmacist, and pharmacologist.	Reduced hospital admissions and outpatient clinic attendances during a follow-up period with a mean duration of 13.9 ± 4 months*.
Toronto, Canada [6]	Clinic with MDT elements	Reviewed 98 patients over a 67-month period.	Face-to-face	Improved BP control, reduced LDL levels, reduced HbA1c, *after a median follow-up of 24 months (10.8–43.3).*
			Cardiologist, nephrologist, endocrinologist, pharmacist. *Available if necessary: dietitian, chiropodist, diabetes nurse educator, ophthalmologist technician.*	
Salford, UK [7]	Clinic with MDT elements	Reviewed 209 patients over 8 months, completing more than 450 appointments.	Face-to-face	*No outcomes reported.*
			Nephrologists with a specialist interest in cardiorenal syndrome, diabetologists, and diabetes specialist nurses; *cardiology input is provided via MDT meetings.*	
Stockholm, Sweden [8]	Clinic with MDT elements	A total of 131 participants were randomized over 35 months, and 71 participants were seen in clinic with MDT elements.	Face-to-face and virtual meetings	Reduced heart failure (HF)-related hospital admissions over a 2-year follow-up (not statistically significant)^1^.
			Patients are first assessed by a specialist nurse, followed by physicians specializing in cardiology/nephrology/endocrinology.	
			The patient is then reviewed and discussed at bi-weekly interdisciplinary meetings, and follow-up is scheduled for 4 weeks after the initial visit.	
Montreal, Canada [9]	Combined clinic (C, R, M)	Reviewed 232 patients over a 41-month period.	Face-to-face Patients are seen by a research nurse and a research doctor. Afterward, the care of each patient is reviewed jointly by a cardiologist, nephrologist, and endocrinologist. The patient is then seen by all three clinicians together to discuss the care plan and answer any questions.	Increased hemoglobin, reduced HbA1c, reduced NT-proBNP, reduced uACR, *observed after a median of 187 days (123–273) from first to last visit.*
Glasgow, UK [10]	Pharmacist-led clinic	Reviewed 225 patients over a 26-month period.	Face-to-face	Increased eGFR 12-months post-intervention.
			Patients are screened in primary care using routinely collected data from electronic health records. An experienced pharmacist then meets with the patient for a 20-minute appointment to discuss medications and care plans to optimize their care.	
Ohio, USA [11]	Health program	Reviewed 426 patients over a 28-month period.	Face-to-face or virtual The team consists of a program administrator; five cardiologists with special interest and training in T2DM management, prevention, and vascular medicine; two nurse coordinators; a dietitian; two certified diabetes care and education specialists; a pharmacist.	Reduced BMI, reduced BP, reduced HbA1c, reduced cholesterol and LDL, reduced triglycerides, *at a median follow-up of 4 months (3–7), with a maximum follow-up of 19 months.*
Various locations, USA [12]	Clinic intervention	Recruited 43 cardiology clinics and randomized 25 to the intervention arm. Recruited 1049 participants and randomized 459 to the intervention group.	Face-to-face and virtual A travelling trio, consisting of an endocrinologist, a cardiologist, and an implementation specialist with clinical nursing experience, initiated the intervention in a cardiology clinic to improve care.	Reduced incidence of the composite event (all-cause mortality or hospitalization for myocardial infarction, stroke, decompensated HF, or urgent revascularization) *after 12 months of follow-up*.

^1^ Study participants were randomized using ballots in sealed envelopes, which did not result in equal group sizes (intervention, n = 73 vs. control, n = 58) (2 participants withdrew from the study after randomization but prior to receiving the intervention). Participants randomized to the intervention group had worse kidney function (eGFR 41.6 ± 13.3 vs. 51.0 ± 17.1; *p* < 0.01) and higher N-terminal pro-B-type natriuretic peptide levels (1760 [889–3923] vs. 971 [261–2535]; *p* < 0.01). * Study compared hospital admissions over the same follow-up duration before the intervention (MDT discussion) and after. BP, blood pressure; LDL, low-density lipoprotein; HbA1c, hemoglobin A1c; NT-proBNP, N-terminal pro–B-type natriuretic peptide; uACR, urinary albumin-to-creatinine ratio; eGFR, estimated glomerular filtration rate; T2DM, type 2 diabetes mellitus; MDT, multidisciplinary team.

These drugs, while associated with long-term benefits, such as reduced rates of hospitalization and death, often have challenging immediate effects, including hyperkalemia and acute rises in creatinine. This often leads to medications being started and stopped because different specialties, operating in silos and with differing priorities, make uncoordinated changes, fragmenting care and delaying effective guideline-directed medical therapy (GDMT) [16]. Notably, these effects increase patient frustration and create challenges for specialty and primary care physicians, as patient journeys are documented across multiple clinic letters that do not cohere into a clear, cohesive plan.

In recognition of this problem, various models of care have been proposed and trialed worldwide to deliver care synchronously across the three specialties: nephrology, endocrinology, and cardiology. However, determining the optimal model that enables cross-specialty coordination, reduces the appointment burden for both patients and clinicians, and improves outcomes across all three systems remains a challenge. Accordingly, this review aims to summarize existing models of care for patients with CKM syndrome and provide a brief discussion of potential adaptations for LMICs.

## 2. Models of Care

Two main care models are currently described in the literature for managing CKM syndrome: clinic-based and multidisciplinary team (MDT)-based models (Table 1).

### 2.1 Clinics

**Fig. 1.**
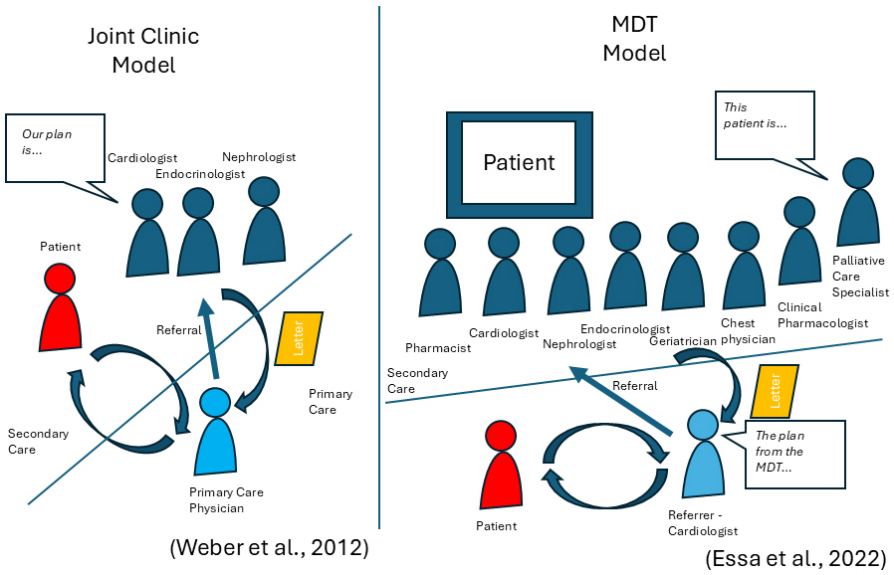
**Diagram of different models of care**. The process of the Joint Clinic Model [4] and the multidisciplinary team (MDT) model [5].

In Vancouver, Canada, a cardio–renal–metabolic clinic was developed and evaluated for effectiveness in a randomized controlled trial (Fig. 1, Ref. [4,5]). A total of 139 patients attending a kidney care clinic and either a heart failure (HF) clinic and/or a diabetes clinic were randomized between July and October 2005. The control arm continued to attend multiple clinics separately, while the intervention arm attended a single integrated, multispecialty care clinic, where patients were seen simultaneously by a nephrologist, a cardiologist, and an endocrinologist. Over a 3-year follow-up period, the integrated multidisciplinary clinic was not inferior to attending separate specialty clinics in preventing hospitalizations. In addition, integrating multiple specialty clinics was estimated to reduce the cost of care by USD 1234 per patient per year compared with attending multiple specialty clinics. Since this trial is now 20 years old, these findings likely underestimate the benefits of a similar clinic today, given the broader range of available medications and interventions, such as SGLT2 inhibitors and GLP-1 agonists.

Similarly, a heart–nephrology–diabetes (HND) clinic was established in Stockholm, Sweden. This clinic combined an initial consultation with a specialist nurse and a follow-up evaluation by a cardiologist, nephrologist, or endocrinologist. Patients were then discussed at fortnightly interdisciplinary meetings to develop comprehensive treatment plans. Follow-up was organized 4–6 weeks later and managed primarily by specialist nurses, with frequent telephone calls. To evaluate the effectiveness of the clinic, a randomized controlled trial was conducted between March 2015 and October 2021, in which 131 patients were randomized to either the integrated HND clinic or standard care, where each patient was seen separately in each specialist clinic [8]. Eligible participants had established CVD, CKD stage 3–4, and diabetes mellitus. After adjustment for baseline differences between groups, HND care clinics tended to reduce the risk of HF-related hospitalizations (hazard ratio (HR) 0.53, 95% confidence interval (CI) 0.28–1.01; *p* = 0.05), and subgroup analysis showed that patients with HF had reduced risk from the intervention (HR 0.55, 95% CI 0.33–0.91; *p* = 0.016). Integrated care also improved physical and social function scores, and self-rated health (*p* = 0.02, *p* = 0.02, and *p* = 0.01, respectively). Following participant feedback, the possibility of crossover from standard care to the integrated clinic after 1 year was introduced and accepted by 51% of the control group. Although these actions complicated the analysis, they also showed strong patient preference for combined care. The crossover would likely strengthen the argument that this clinic is effective, as it would likely underpower the study, which reported statistically significant improvements in physical and social function scores, as well as a reduced risk of hospitalization among participants with HF.

A similar model, the Cardiac and Renal Endocrine (CaRE) clinic, was developed in Toronto, Canada [6], as a monthly, multidisciplinary, interprofessional service accepting referrals for patients with type 2 diabetes and co-existing renal disease and/or CVD. This clinic not only combined cardiology, nephrology, and endocrinology clinics into a single consultation, but also included other disciplines, such as chiropodists, ophthalmologists, dietitians, and pharmacists, to support clinic attendees. The effectiveness of this setup was evaluated in a retrospective cohort study from July 2014 to February 2020. Of 118 patients referred, 74 had data available for both their first and last clinic visits and were included in the analysis. The study found a higher uptake of SGLT-2 inhibitors (35.1% vs. 4.1%; *p* < 0.01), GLP-1 agonists (13.5% vs. 4.1%; *p* = 0.02), and statins (93.2% vs. 81.1%; *p* = 0.01). This was associated with significant improvements in hemoglobin A1c (HbA1c) (7.5% vs. 7.1%; *p* = 0.02), low-density lipoprotein (LDL) cholesterol (1.9 mmol/L vs. 1.5 mmol/L; *p* < 0.01), and the proportion of patients achieving the required blood pressure (BP) target (52.7% vs. 36.5%; *p* = 0.04). No change was noted in body mass index (BMI) (29.7 kg/m^2^ vs. 29.6 kg/m^2^; *p* = 0.15). Nonetheless, this study is limited by the small sample size, which not only reduces statistical power but also suggests the model may be challenging to replicate at scale, given that only 74 of 118 referred participants had complete data over nearly 6 years.

More recently, a CKM clinic was also developed in Montreal, Canada [17]. Patients were first assessed by a specialist nurse, who triaged the patients to the most appropriate specialist, such as a cardiologist for those with HF. The specialist physician then discussed the case with the other specialty physicians, in this case nephrology and endocrinology, to reach a consensus care plan. All three specialties then saw the patient together to address questions, and two further appointments were scheduled to optimize medications. The effectiveness of the clinic was evaluated in a retrospective cohort study of 232 patients seen between October 2020 and March 2024 [9]. The use of GDMT increased significantly between baseline and the last clinic visit, with an increase in the use of SGLT2 inhibitors (46% vs. 87%; *p* < 0.01), GLP-1 agonists (10% vs. 48%; *p* < 0.01), renin–angiotensin–aldosterone system (RAAS) inhibitors (77% vs. 91%; *p* < 0.01), and mineralocorticoid receptor antagonists (25% vs. 44%; *p* < 0.01). Overall, there was a reduction in the use of non-prognostically beneficial diabetes drugs, such as sulfonylureas (27% vs. 21%; *p* = 0.04), dipeptidyl peptidase-4 (DPP-4) inhibitors (34% vs. 15%; *p* < 0.01), and insulin (27% vs. 17%; *p* < 0.01). These therapeutic changes were accompanied by improvements in laboratory biomarkers, with decreases in N-terminal pro–B-type natriuretic peptide (NT-proBNP) (–469 pg/mL, 95% CI –750 to –189; *p* < 0.01), HbA1c (–0.6%, 95% CI –1.0% to –0.3%; *p* < 0.01), and urine albumin–creatinine ratio (–215 mg/g, 95% CI –422 to –9; *p* = 0.04) between baseline and the last clinic visit. This study shows that the clinic applies guideline-directed therapy effectively across all three specialties for these patients but struggles to demonstrate superiority over conventional therapy due to the lack of a comparator.

### 2.2 Innovation Beyond Clinics

In a different approach, a large MDT was established in Liverpool, United Kingdom, to manage patients with multiple comorbidities and HF [5]. A monthly multispecialty MDT meeting was conducted via online videoconferencing. This meeting brought together HF cardiologists from secondary and tertiary care, HF nurses, a nephrologist, an endocrinologist, a geriatrician, a chest physician, a palliative care specialist, a pharmacist, and a clinical pharmacologist. All MDT members discussed patients and provided input on optimizing GDMT for HF, CKD, and diabetes control, as well as on complex chronic respiratory pathologies, fall risk assessment, cognitive dysfunction, and advanced care planning. The impact of this multispecialty, multidisciplinary input was evaluated between January 2020 and June 2021 and compared with an observational retrospective cohort study of 334 patients. The study found that the MDT successfully optimized diabetes management (HbA1c pre-MDT 68 ± 11 mmol/mol vs. post-MDT 61 ± 9 mmol/mol; *p* < 0.001), initiated RAAS inhibitors in HF with reduced ejection fraction (HFrEF) with advanced CKD (9% pre- vs. 71% post-MDT; *p* < 0.001), and reduced all-cause hospitalizations (pre-MDT 1.1 ± 0.4 vs. 0.6 ± 0.1 post-MDT; *p* < 0.01). The introduction of the MDT was estimated to result in total cost savings to the healthcare system of approximately USD 890,000, or USD 2600 per patient. This study compared pre- and post-MDT outcomes among participants to assess the impact of the MDT. However, there was no concurrent standard-of-care comparator group to determine whether the MDT enhanced treatment outcomes.

Further north in Glasgow, a different service model was developed. Clinical pharmacists based in primary care practices reviewed the electronic health records of patients and developed individualized care plans [10]. These plans were then discussed with patients to optimize comorbidity treatment and minimize potentially harmful medications, such as nephrotoxic agents. Although this was a prospective intervention scoping study and all analyses were conducted for hypothesis-generating purposes, the study showed a minor but statistically significant improvement in eGFR 12 months after the intervention (2.9 mL/min/1.73 m^2^; 95% CI, 1.41–4.40; *p* < 0.01); while statistically significant, this result is not clinically significant. Future fully powered trials could address this by using eGFR slope as an outcome measure or by extending the follow-up period.

Currently, all interventions require patients to attend a clinic or be referred to an MDT. Instead, a team in Cleveland, Ohio, USA, set up a program to bring a multi-specialty clinic to patients. Using a combination of in-person and virtual visits, the clinic brings together five cardiologists, a program administrator, a nurse coordinator, a dietitian who is also a certified diabetes care and education specialist, and a pharmacist. After an initial visit to determine the overall cardiovascular risk of each patient, the team devises a comprehensive lifestyle and pharmacological intervention plan. Patients are then supported by the team, including insurance, pharmacy, and assistance programs staff, to obtain the medication(s) in the most affordable way possible. Between May 2020 and September 2022, 426 patients were enrolled in the CINEMA program. Over a median follow-up of 4 months (interquartile range (IQR) 3–7 months), using a linear mixed regression model, CINEMA is estimated to have reduced the BMI by 0.86 kg/m^2^ (95% CI 0.68–1.05; *p* < 0.01) and HbA1c by 0.47% (95% CI 0.35–0.59; *p* < 0.01) [11]. A comprehensive assessment that incorporates multiple specialties, along with support from insurance and assistance programs, appears helpful to patients. However, these factors are mainly specific to the United States healthcare provision context and may not be directly transferable to systems without adaptation.

**Fig. 2.**
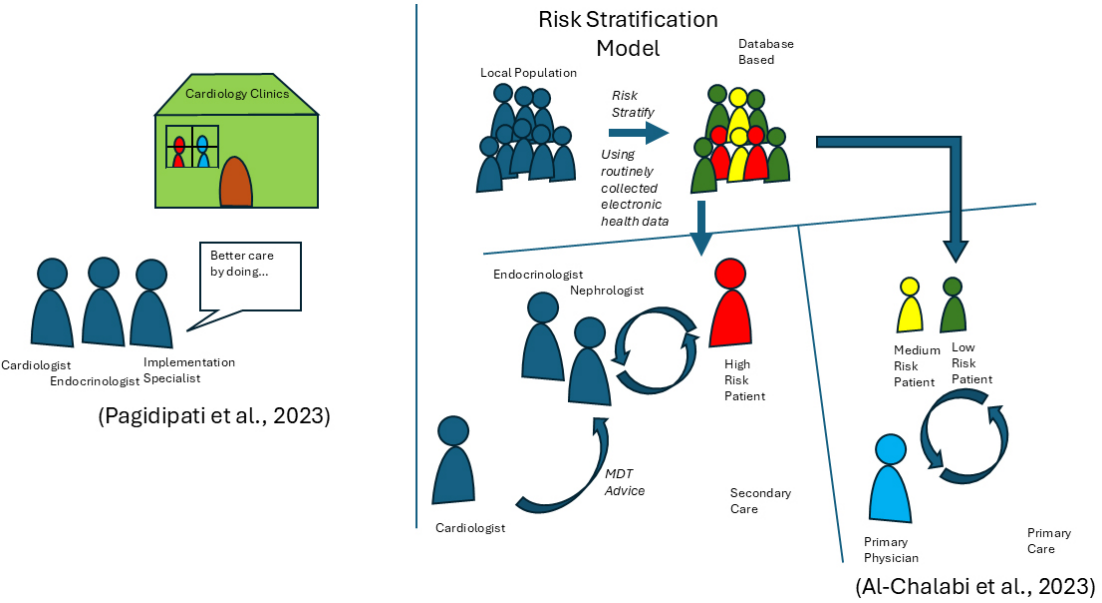
**Diagram of less-conventional models of care**. The models of Pagidipati et al. [12], which improve cardiology clinics, and Al-Chalabi et al. [7], which utilize digital health to screen patients.

Not all interventions focused solely on patients. Pagidipati et al. [12] showed that a trio comprising cardiologists, endocrinologists, and an implementation specialist could improve cardiology clinics across the United States, increasing prescribing rates of high-intensity statins, angiotensin converting enzyme (ACE) inhibitors or angiotensin-receptor blockers (ARB), and SGLT2 inhibitors or GLP-1 agonists in a clustered randomized controlled trial. Cardiology clinics randomized to the intervention arm received clinic-specific analyses of barriers to evidence-based care, development of local interdisciplinary pathways, support for coordination of care among clinicians within the local clinics, clinician education on current practice guidelines, a standardized monthly audit and feedback of quality metrics, and educational materials for patients (Fig. 2, Ref. [7,12]). The tailored nature of the clinic-level barriers and the specific solutions provided by the trio generated heterogeneity in results and may limit the generalizability and ease of replication of this model in other settings.

In Salford, UK, an outpatient improvement program was established that used modern electronic health records to analyze routinely collected healthcare data and produce a dashboard to identify and risk-stratify patients [7]. Following this initial filtering process, high-risk patients were invited to a CKM clinic, where a nephrologist with cardiovascular expertise, a diabetologist, and a diabetes specialist nurse assessed each patient. Cardiology input was provided via MDT meetings. According to a brief report, this multispecialty clinic provided care to 209 patients in the first 8 months. Unfortunately, this short report does not include any clinical outcome data. Analysis of clinical outcomes is planned for 2025 and is expected to clarify the effectiveness of this novel intervention.

At St George’s Hospital, London, authors D.B., L.A., and M.C. have established a CKM clinic in which referred patients are seen jointly by an endocrinologist, a cardiologist, and a nephrologist, enabling a comprehensive management plan and opportunities for patients to address questions with all three specialties. This clinic primarily serves patients with CKM stage 4. In response to this, our clinic began visiting individual primary health surgeries to discuss patients with CKM stage 2–4 and to formulate a comprehensive medical plan to be implemented by the associated primary health physician. At present, we have managed 150 patients in this pilot scheme. Initial audits showed that clinic-generated plans included input from at least two specialties in 67.6% of cases and from all three specialties in 55.9% of cases. The most common interventions were optimization of HF and CKD medications (68.5%), optimization of glycemic control (62.2%), and obesity management (62.2%).

In South West London, a multispecialty clinic for CKD–hemodialysis (HD) patients, staffed by cardiologists and nephrologists, has been shown to be beneficial for optimizing RAAS inhibitor therapy and anemia management [18]. However, much more can be achieved to reduce hospitalizations in this vulnerable group of patients with renal impairment and HF [19].

Collectively, these studies show that there are innovative approaches to detecting, assessing, and treating these complex patients within individual health systems. However, these approaches share similar limitations in how each navigates and utilizes the intrinsic characteristics of the associated health systems to benefit individual patients and mitigate system-level shortcomings, such as the CINEMA program having specialists to navigate the difficulties around health insurance in the United States, or the Salford study that utilizes the ubiquitous nature of primary care services in the United Kingdom. These context-specific features make such models difficult to replicate in countries that lack similar infrastructure.

Moreover, these schemes are resource-intensive and are therefore likely to be difficult to implement in LMICs.

### 2.3 CKM Care in Low- and Middle-Income Countries

CKM syndrome affects a disproportionately higher proportion of the population in LMICs. The global prevalence of CKD alone is about 788 million, with the highest rates observed in LMICs, at 15.2% and 16.3%, respectively, compared with 10.8% in high-income countries [20]. Of the 589 million people with diabetes worldwide, the largest numbers live in Southeast Asia (107 million) and the West Pacific Rim (215 million). Currently, an estimated 4 in 5 patients with T2DM live in LMICs [21]. In 2023, 1.5 billion people were living with metabolic syndrome, again with the highest prevalence in LMICs [22]. The high prevalence of metabolic syndrome, diabetes, and CKD results in a high incidence of CVD in LMICs. In 2023, CVD was the leading cause of disability-adjusted life years (DALYs) and death globally, accounting for 19.2 million deaths and 437 million DALYs. Age-standardized CVD DALY rates were highest in low- and low–middle sociodemographic index (SDI) regions and lowest in high-SDI regions [23].

Low SDI countries spend the least on healthcare; hence, access to preventive care for CKM syndrome is limited, resulting in poor outcomes, such as disability, hospital admissions, and death. In addition, low health literacy, public and political willingness to tackle disease, combined with food insecurity, unemployment, and poverty, results in poor care for patients with CKM syndrome, particularly in low-income regions. Therefore, addressing CKM syndrome in this context will require a pragmatic approach, grounded in innovative, simple-to-implement, technology-assisted strategies as outlined below.

First, expanding access to diagnostics is critical for early identification of at-risk individuals. A simple, low-cost diagnostic bundle in primary care, comprising basic anthropometric measurements (height, weight, and waist circumference), urine dipstick testing, lipid profile, fasting blood glucose, and serum creatinine, can facilitate early diagnosis and effective risk stratification of CKM syndrome. Earlier identification allows timely intervention, reducing progression to advanced disease that requires expensive specialist care.

Second, increasing access to care can be achieved by extending the healthcare workforce beyond physicians. Education and training of allied healthcare professionals, community health workers, and lay health providers in the basic identification and management of CKM syndrome can substantially expand service capacity. Digitalization, through mobile phones and digital health platforms, can further amplify outreach, support risk identification, and enable guideline-driven management at scale. Community-based digital initiatives, such as large population programs implemented in Indonesia, demonstrate the feasibility of this approach in rural, resource-limited settings [24].

Third, strengthening the confidence of physicians in managing CKM syndrome in primary care can reduce the use of high-cost specialist services. With appropriate education and clinical pathways, primary care physicians can safely manage key components of CKM syndrome, including BP control to guideline-recommended targets and the use of evidence-based therapies such as ACE inhibitors, SGLT2 inhibitors, and GLP-1 agonists. Enhancing primary care capability reduces care costs for individuals, alleviates specialist burden, and increases volume of care provided by the system while maintaining quality of care.

Fourth, community-wide campaigns focused on CKM syndrome, the associated adverse effects, and management are required to improve health literacy and political willingness to tackle the rising burden of this syndrome.

### 2.4 Beyond the Horizon 

Apart from clinicians managing patients, there has been increasing recognition of the importance of patient self-management, paralleling the revolution in diabetes care brought about by continuous glucose monitoring (CGM) systems [25].

Firstly, there has been an increasing use of commercially available wearable devices, such as Fitbits, in clinical trials. For example, a randomized controlled trial of patients with HF evaluated the relationship between daily step count and health status, as assessed by the Kansas City Cardiomyopathy Questionnaire (KCCQ) [26]. The study found that higher step counts were associated with significantly higher KCCQ scores for total symptoms (*p* < 0.001) and physical limitation (*p* < 0.001) at baseline. Moreover, changes in daily step count were associated with significant alterations in KCCQ scores for total symptoms (*p* = 0.004) and physical limitation (*p* = 0.003) from baseline to 12 weeks. These findings highlight the potential uses of commercially available wearable devices for monitoring and improving cardiovascular outcomes.

In addition to commercially available products, there has also been an effort to use custom-built wearable technologies to help guide clinical decision-making. A randomized controlled trial showed that a wearable device, capable of measuring ECG signals via two electrodes and detecting thoracic fluid levels using low-energy electromagnetic waves, helped guide the management of patients with acute decompensated HF following hospital discharge [27]. The study found that using data from the HF monitoring system wearable device to guide HF management reduced the 90-day HF readmission rate by 38% (HR 0.62; *p* = 0.03), with an absolute reduction of 7%. The number needed to treat to prevent hospitalization with HF was 14.3. In addition, patients in the intervention group reported an average 12-point improvement in quality of life compared with the control group (*p* < 0.01). These results suggest significant benefits of remote monitoring devices in the management of HF.

In addition to wearable technologies, point-of-care (POC) testing has attracted renewed research interest, particularly for screening and monitoring CKD and for adjusting renal medication doses in community settings [28]. The value of using POC testing for community screening was demonstrated by the First Nations Community-Based Screening to Improve Kidney Health and Prevent Dialysis (FINISHED) trial, which aimed to identify individuals with diabetes, hypertension, and CKD in rural and remote regions of Canada [29]. Among 1346 adults screened, 25.5% had CKD, with a higher prevalence in remote communities accessible only by air (34.4%) than in those accessible by road (17.6%), highlighting the value of simple, easily accessible screening tools. In another example, a Spanish study evaluated the effectiveness of POC creatinine testing used in community pharmacy settings for flagging nephrotoxic drugs for adjustment or withdrawal by primary care physicians [30]. In a cohort of 87 patients taking 635 medications, POC creatinine testing identified 50 (7.9%) medications affected by impaired renal function, recommending dose adjustment for 31 and withdrawal for 19. Of these, primary care physicians accepted six (19%) recommendations for dose adjustment and eight (42%) for withdrawal, demonstrating the utility of POC testing for medication adjustment in the community.

Collectively, these new technologies may generate rich data to support better clinical management and risk prediction, and enhance patient autonomy by enabling self-management steps, such as adjusting furosemide doses in a manner analogous to CGM-guided insulin adjustment in diabetes. These remote monitoring tools may also pave the way for managing HF patients at home, as demonstrated by the “*Managing heart failure @home*” initiative in the UK [31].

### 2.5 Future Directions

**Fig. 3.**
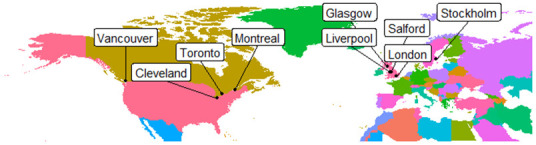
**Map of models of care described in this review**. A map of North America and Europe illustrates that all models of care described in this review are located in high-income countries in these regions. Original figure generated with R https://www.r-project.org/ and ggplot2 https://ggplot2.tidyverse.org/.

Fig. 3 shows a distinct problem: all current published CKM initiatives have been based on high-income countries in Europe and North America. This is concerning because epidemiological studies have shown a similar risk of CKM in large populous nations such as India [32] and China [33]. The problem is further exacerbated by risk calculators such as the PREVENT [34], which has not been validated in South Asian populations. Therefore, global initiatives and guidelines must consider local conditions.

## 3. Conclusion

The optimal management approach for CKM syndrome has yet to be defined. Multiple initiatives worldwide aim to identify the most effective and patient-friendly strategies for managing patients with CKM syndrome, whether in a clinic setting with multiple specialties, at an MDT meeting, or through novel technologies that facilitate patient self-management. The most effective intervention will likely combine clinic-based specialist care for high-risk patients and self-management supported by novel technologies for low-risk patients, to reduce disease progression and provide high volumes of care. However, each solution will need to be tailored to the individual health system.

Nonetheless, further research is needed to determine whether an optimal model of care for CKM syndrome can be defined, which will improve morbidity and mortality outcomes and enhance quality of life for patients.

## Abbreviations

CKD, chronic kidney disease; CKM, cardio–kidney–metabolic; CVD, cardiovascular disease; GLP-1, glucagon-like peptide 1; HF, heart failure; KCCQ, Kansas City Cardiomyopathy Questionnaire; RAAS, renin–angiotensin–aldosterone system; SGLT-2, sodium–glucose co-transporter 2.

## References

[1] Sebastian SA, Padda I, Johal G (2024). Cardiovascular-Kidney-Metabolic (CKM) syndrome: A state-of-the-art review. *Current Problems in Cardiology*.

[2] Narula J, Butler J, Chothia Y, Bannerjee D, Jarraya F, Ulasi I (2025). Optimising Access to Care for Patients with Heart and Kidney Diseases: A World Heart Federation and International Society of Nephrology White Paper. *Global Heart*.

[3] Ndumele CE, Rangaswami J, Chow SL, Neeland IJ, Tuttle KR, Khan SS (2023). Cardiovascular-Kidney-Metabolic Health: A Presidential Advisory From the American Heart Association. *Circulation*.

[4] Weber C, Beaulieu M, Djurdjev O, Er L, Taylor P, Ignaszewski A (2012). Towards rational approaches of health care utilization in complex patients: an exploratory randomized trial comparing a novel combined clinic to multiple specialty clinics in patients with renal disease-cardiovascular disease-diabetes. *Nephrology, Dialysis, Transplantation*.

[5] Essa H, Walker L, Mohee K, Oguguo C, Douglas H, Kahn M (2022). Multispecialty multidisciplinary input into comorbidities along with treatment optimisation in heart failure reduces hospitalisation and clinic attendance. *Open Heart*.

[6] Dubrofsky L, Lee JF, Hajimirzarahimshirazi P, Liu H, Weisman A, Lawler PR (2022). A Unique Multi- and Interdisciplinary Cardiology-Renal-Endocrine Clinic: A Description and Assessment of Outcomes. *Canadian Journal of Kidney Health and Disease*.

[7] Al-Chalabi S, Alderson H, Garratt N, Green D, Kalra PA, Ritchie J (2023). Improving outpatient clinic experience: the future of chronic kidney disease care and associated multimorbidity. *BMJ Open Quality*.

[8] Evén G, Stenfors T, Jacobson SH, Jernberg T, Franzén-Dahlin Å, Jäghult S (2024). Integrated, person-centred care for patients with complex cardiovascular disease, diabetes mellitus and chronic kidney disease: a randomized trial. *Clinical Kidney Journal*.

[9] Marques P, Mavrakanas TA, Guida J, Gédéon T, Emami A, Alaamiri A (2024). Utilizing synchronous care to improve cardiovascular and renal health among patients with type 2 diabetes: Proof-of-concept results from the DECIDE-CV clinical programme. *Diabetes, Obesity & Metabolism*.

[10] Ramos T, Verma A, Speirits I, Zhang L, McInally J, McShane C (2025). Evaluating a pharmacist-led cardio-renal-metabolic service to reduce healthcare inequities in a socioeconomically deprived population: a prospective intervention study. *International Journal of Clinical Pharmacy*.

[11] Neeland IJ, Arafah A, Bourges-Sevenier B, Dazard JE, Albar Z, Landskroner Z (2023). Second-year results from CINEMA: A novel, patient-centered, team-based intervention for patients with Type 2 diabetes or prediabetes at high cardiovascular risk. *American Journal of Preventive Cardiology*.

[12] Pagidipati NJ, Nelson AJ, Kaltenbach LA, Leyva M, McGuire DK, Pop-Busui R (2023). Coordinated Care to Optimize Cardiovascular Preventive Therapies in Type 2 Diabetes: A Randomized Clinical Trial. *JAMA*.

[13] Anker SD, Butler J, Filippatos G, Ferreira JP, Bocchi E, Böhm M (2021). Empagliflozin in Heart Failure with a Preserved Ejection Fraction. *The New England Journal of Medicine*.

[14] Kosiborod MN, Abildstrøm SZ, Borlaug BA, Butler J, Rasmussen S, Davies M (2023). Semaglutide in Patients with Heart Failure with Preserved Ejection Fraction and Obesity. *The New England Journal of Medicine*.

[15] Solomon SD, McMurray JJV, Vaduganathan M, Claggett B, Jhund PS, Desai AS (2024). Finerenone in Heart Failure with Mildly Reduced or Preserved Ejection Fraction. *The New England Journal of Medicine*.

[16] Rangaswami J, Tuttle K, Vaduganathan M (2020). Cardio-Renal-Metabolic Care Models: Toward Achieving Effective Interdisciplinary Care. *Circulation. Cardiovascular Quality and Outcomes*.

[17] Alqahtani M, Ganni E, Mavrakanas T, Tsoukas M, Peters T, Suri R (2022). Synchronous Health Care Delivery for the Optimization of Cardiovascular and Renal Care in Patients with Type 2 Diabetes. *Current Cardiology Reports*.

[18] Nguyen M, Rumjaun S, Lowe-Jones R, Ster IC, Rosano G, Anderson L (2020). Management and outcomes of heart failure patients with CKD: experience from an inter-disciplinary clinic. *ESC Heart Failure*.

[19] Parmar S, Lopez T, Shah R, Murphy D, Warrens H, Khairallah M (2025). Risk of Hospital Admissions and Death in Patients with Heart Failure and Chronic Kidney Disease: Findings from a Novel Multidisciplinary Clinic. *Cardiorenal Medicine*.

[20] Mark PB, Stafford LK, Grams ME, Aalruz H, Abd ElHafeez S, Abdelgalil AA (2025). Global, regional, and national burden of chronic kidney disease in adults, 1990-2023, and its attributable risk factors: a systematic analysis for the Global Burden of Disease Study 2023. *The Lancet*.

[21] International Diabetes Federation IDF Diabetes Atlas 2025. https://diabetesatlas.org/resources/idf-diabetes-atlas-2025/.

[22] Noubiap JJ, Nansseu JR, Nyaga UF, Ndoadoumgue AL, Ngouo AT, Tounouga DN (2025). Worldwide trends in metabolic syndrome from 2000 to 2023: a systematic review and modelling analysis. *Nature Communications*.

[23] Global Burden of Cardiovascular Diseases and Risks 2023 Collaborators (2025). Global, Regional, and National Burden of Cardiovascular Diseases and Risk Factors in 204 Countries and Territories, 1990-2023. *Journal of the American College of Cardiology*.

[24] Patel A, Praveen D, Maharani A, Oceandy D, Pilard Q, Kohli MPS (2019). Association of Multifaceted Mobile Technology-Enabled Primary Care Intervention With Cardiovascular Disease Risk Management in Rural Indonesia. *JAMA Cardiology*.

[25] Martens T, Beck RW, Bailey R, Ruedy KJ, Calhoun P, Peters AL (2021). Effect of Continuous Glucose Monitoring on Glycemic Control in Patients With Type 2 Diabetes Treated With Basal Insulin: A Randomized Clinical Trial. *JAMA*.

[26] Golbus JR, Gosch K, Birmingham MC, Butler J, Lingvay I, Lanfear DE (2023). Association Between Wearable Device Measured Activity and Patient-Reported Outcomes for Heart Failure. *JACC. Heart Failure*.

[27] Boehmer JP, Cremer S, Abo-Auda WS, Stokes DR, Hadi A, McCann PJ (2024). Impact of a Novel Wearable Sensor on Heart Failure Rehospitalization: An Open-Label Concurrent-Control Clinical Trial. *JACC. Heart Failure*.

[28] Gama RM, Nebres D, Bramham K (2024). Community Point of Care Testing in Diagnosing and Managing Chronic Kidney Disease. *Diagnostics*.

[29] Komenda P, Lavallee B, Ferguson TW, Tangri N, Chartrand C, McLeod L (2016). The Prevalence of CKD in Rural Canadian Indigenous Peoples: Results From the First Nations Community Based Screening to Improve Kidney Health and Prevent Dialysis (FINISHED) Screen, Triage, and Treat Program. *American Journal of Kidney Diseases*.

[30] Escribá-Martí G, Cámara-Ramos I, Climent-Catalá MT, Escudero-Quesada V, Salar-Ibáñez L (2022). Pharmaceutical care program for patients with chronic kidney disease in the community pharmacy: Detection of nephrotoxic drugs and dose adjustment. Viability study. *PLoS ONE*.

[31] NHS England (2025). Managing heart failure @home. https://www.england.nhs.uk/nhs-at-home/managing-heart-failure-at-home/.

[32] Vijayaraghavan K, McCullough PA, Singh B, Gupta M, Enas E, Mohan V (2019). Cardiometabolic-Renal Disease in South Asians: Consensus Recommendations from the Cardio Renal Society of America. *Cardiorenal Medicine*.

[33] Huang Q, Li Y, Zhou X, Zou X, Ji L (2025). Prevalence of the Cardiovascular-Kidney-Metabolic Syndrome in 3 Chinese Cohorts. *JACC. Asia*.

[34] Khan SS, Matsushita K, Sang Y, Ballew SH, Grams ME, Surapaneni A (2024). Development and Validation of the American Heart Association’s PREVENT Equations. *Circulation*.

